# Machine Learning Prediction of Road Performance of Cold Recycled Mix Asphalt with Genetic Algorithm Hyperparameter Optimization

**DOI:** 10.3390/ma18245635

**Published:** 2025-12-15

**Authors:** Zongyuan Wu, Shiming Li, Decai Wang, Mengxin Qiu, Chenze Fang, Jingyu Yang, Hongjia Tang

**Affiliations:** 1 School of Civil Engineering and Communication, North China University of Water Resources and Electric Power, Zhengzhou 450045, China; wuzongyuan@ncwu.edu.cn (Z.W.); lishiming@ncwu.edu.cn (S.L.);; 2 Provincial Engineering Research Center, New Energy Vehicle Intelligent Control and Simulation Test Technology of Sichuan, Chengdu 610039, China

**Keywords:** cold recycled mix asphalt, machine learning prediction, genetic algorithm, SHAP interpretation, road performance

## Abstract

With the rapid expansion and aging of global road networks, cold recycled mix asphalt (CRMA) has gained significant attention as a sustainable pavement rehabilitation technology. However, the road performance of CRMA is highly sensitive to material composition and curing conditions, making accurate performance prediction challenging. This study develops machine learning (ML) models to predict two critical performance indicators: dynamic stability (DS) for high-temperature stability and indirect tensile strength (ITS) for low-temperature crack resistance. Four ML algorithms, Artificial Neural Network (ANN), Extreme Gradient Boosting (XGBoost), Random Forest (RF), and Support Vector Regression (SVR), were trained on a comprehensive dataset of 436 samples. A genetic algorithm (GA) was employed to optimize model hyperparameters, significantly enhancing prediction accuracy and robustness. The SHAP method was further applied to interpret model outputs and identify key influencing factors. Results demonstrate that GA-optimized XGBoost achieved the highest predictive performance for both dynamic stability (DS) and indirect tensile strength (ITS), with R^2^ values of 0.9793 and 0.9694, respectively. Curing temperature, RAP content, and curing time were identified as the most influential factors. This study provides an accurate and interpretable ML-based framework for CRMA performance prediction, facilitating optimized mix design for pavement construction and maintenance.

## 1. Introduction

With the rapid expansion and aging of global road networks, asphalt pavements have entered a critical stage of large-scale maintenance and renovation [1,2]. In response to the dual demands of resource conservation and environmental protection [3,4,5], asphalt pavement recycling technology has emerged as a research hotspot, enabling the reuse of Reclaimed Asphalt Pavement (RAP). Among recycling technologies, cold recycled mix asphalt (CRMA) stands out compared to hot recycling: it eliminates high-temperature mixing and compaction, thereby reducing energy consumption by 30~50% and lowering pollutant emissions [6,7]. CRMA is further categorized into plant-mixed and in-place cold recycling, with plant-mixed technology dominating engineering applications due to its ability to ensure mixture consistency and long-term durability.

Emulsified asphalt is the primary binder for CRMA, favored for its strong aggregate adhesion, excellent moisture resistance, simple preparation process, and low production cost. However, the road performance of CRMA (e.g., high-temperature stability, rut resistance, low-temperature crack resistance, and moisture stability) is highly sensitive to raw material properties and construction conditions. Key influencing factors include RAP content, virgin aggregate gradation, emulsified asphalt dosage, filler type, curing time, and curing temperature [8,9]. For instance, Jing et al. [10] demonstrated that the fusion degree between virgin asphalt and aged asphalt in RAP directly determines the high-temperature and water stability of CRMA, while Lyu et al. [11] found that CRMA exhibits 22.5% higher flow value attenuation and 53.5% lower fracture energy than new asphalt mixtures after secondary aging, highlighting the need for accurate performance prediction to ensure long-term serviceability.

The road performance of CRMA, particularly its high-temperature stability characterized by dynamic stability (DS) and low-temperature crack resistance characterized by indirect tensile strength (ITS), is influenced by a complex array of factors. Research indicates that RAP content, curing conditions, aggregate gradation and type, filler type, compaction method, and water–cement ratio all play significant and often interactive roles [12,13,14,15,16,17,18,19,20,21,22,23,24,25,26,27,28,29,30,31,32,33,34,35,36,37,38]. For example, RAP content influences the aggregate skeleton and aged asphalt stiffness, with optimal performance observed within specific ranges [12,13,14,15,16]. Curing time and temperature are critical for strength development through demulsification, water evaporation, and hydration [17,18,19,20]. Aggregate gradation dictates the internal structure and load-bearing capacity [21,22,23], while the surface properties of aggregates affect adhesion [24]. The choice of filler, such as cement or lime, significantly modifies mastic properties and early strength but can increase brittleness [25,26,27,28,29]. The compaction method determines the final density and simulation of field conditions [32,33,34,35], and the water–cement ratio is crucial for workability and the avoidance of excessive porosity [36,37,38]. This complex, multi-factorial sensitivity makes accurate performance prediction a significant challenge, driving the search for advanced modeling approaches.

Traditional CRMA design and performance evaluation rely on the Marshall method, which suffers from critical limitations: its vertical impact compaction mechanism fails to simulate the horizontal shear forces encountered in field construction, leading to insufficient specimen densification and excessive emulsified asphalt usage. Additionally, the Marshall method depends on empirical parameters and lacks quantitative assessment of key mechanical indicators such as shear strength, cohesion, and internal friction angle, making it unable to comprehensively predict the long-term performance of CRMA. These shortcomings have driven the search for data-driven approaches to enhance prediction accuracy and efficiency.

In recent years, machine learning (ML) has emerged as a powerful tool for solving complex nonlinear problems in pavement engineering, outperforming traditional empirical models by capturing intricate relationships between input features and performance indicators [39,40]. Atakan et al. [41] used Random Forest (RF) to predict hot-mix asphalt (HMA) strength, integrating aggregate surface area and gradation characteristics to achieve higher accuracy than empirical methods. Majidifard et al. [42] proposed hybrid models including Gene Expression Programming (GEP) and Artificial Neural Network (ANN)/Simulated Annealing (SA) to predict asphalt mixture fracture energy, demonstrating hybrid ML’s potential for complex failure mechanisms. Fakhri et al. [43] investigated the application of meta-heuristic algorithms based on XGBoost to predict the effective fracture toughness of concrete.

To address the “black box” limitation of ML, explainable AI tools such as SHapley Additive exPlanations (SHAP) have been introduced. AL-Jarazi et al. [44] used SHAP to interpret interlayer shear strength predictions from ANN and RF models, identifying aggregate roughness and asphalt viscosity as key drivers to bridge the gap between data-driven outputs and mechanistic insights. While ML models exhibit considerable potential for predicting the road performance of asphalt mixtures, their predictive accuracy and robustness are highly dependent on hyperparameter tuning. This critical step directly affects the ability of models to capture complex nonlinear relationships between input features and performance indicators. As a bio-inspired optimization technique, Genetic Algorithm (GA) has been validated to enhance the robustness of ML models by efficiently searching for globally optimal hyperparameter combinations, thereby addressing the limitations of local optimization. The GA approach was chosen for its ability to perform a comprehensive search in the hyperparameter space, facilitating an in-depth analysis of configurations that enhance agent success. However, this effective optimization method has rarely been applied to the development of ML models for predicting the road performance of CRMA. Most existing studies in this field still rely on manual tuning or simple heuristic methods to determine hyperparameters. Such approaches often fail to identify the optimal parameter space, resulting in sub-optimal model accuracy and poor generalization capabilities when faced with diverse engineering scenarios.

Existing literature reveals three key research gaps that hinder the advancement of ML-based CRMA performance prediction. First, there is a lack of systematic comparison of multiple mainstream ML algorithms for CRMA road performance prediction. While algorithms such as ANN, XGBoost, RF, and Support Vector Regression (SVR) have been widely used in predicting the performance of HMA and demonstrated their respective advantages, no studies have specifically targeted CRMA to evaluate the adaptability and predictive performance of these four algorithms across different core road performance indicators. This gap leaves engineering practitioners and researchers uncertain about which algorithm is most suitable for specific CRMA performance prediction tasks, limiting the targeted application of ML in CRMA design. Second, inadequate hyperparameter optimization persists in existing CRMA-related ML models. As mentioned earlier, GA has not been widely adopted in CRMA performance prediction. The excessive dependence on default parameters or simple tuning methods leads to inadequate utilization of the potential of ML models, further restricting their predictive accuracy and robustness. Third, although SHAP method has been successfully applied to interpret ML models for HMA, it has not been fully extended to CRMA performance prediction models. This deficiency makes it challenging to clarify the quantitative contribution of key influencing factors to the road performance of CRMA. It hinders the establishment of a mechanistic link between model outputs and the internal material behavior of CRMA and reducing the practical guiding value of ML models for CRMA mix design optimization.

To address the identified research gaps and achieve the goal of establishing accurate and interpretable predictive tools for the road performance of CRMA, this study follows a systematic workflow centered on data-driven modeling with hyperparameter optimization and mechanistic interpretation. First, a comprehensive and multi-source dataset is constructed to lay a reliable foundation for model training and validation. Four ML models involving ANN, XGBoost, RF, and SVR, are subsequently developed for performance prediction. The GA is employed to optimize the hyperparameters of each model to enhance model robustness and avoid suboptimal performance caused by default parameters. On this basis, predicted values of key performance indicators are compared with ground truth to evaluate the predictive performance of the optimized models. The SHAP method is further applied to analyze feature importance and marginal effects. Overall, this study aims to develop a fast, accurate, and interpretable predictive framework for CRMA road performance, with a particular focus on two key performance indicators: DS for high-temperature performance and ITS for low-temperature crack resistance. By integrating genetic algorithm optimization and SHAP-based interpretability, this research seeks to provide reliable tools for optimizing CRMA mix design and promoting the sustainable application of cold recycling technology in pavement engineering.

## 2. Dataset

Current studies rarely incorporate reliable datasets that encompass the key common features governing the performance of CRMA. However, a high-quality comprehensive dataset is essential for developing CRMA pavement performance prediction models. Machine learning models rely on datasets containing authentic and desired input-output variables, derived from a large number of samples obtained through previous laboratory experiments. These data samples serve as the foundation for training and establishing machine learning models. This study establishes a novel database tailored for training machine learning models. This dataset integrates 316 sample data extracted from peer-reviewed literature [45,46,47,48,49,50,51,52,53,54,55,56,57,58,59,60,61,62,63,64,65,66,67,68,69,70,71,72,73], and 120 sample data from the original laboratory experimental data generated by the authors [74], totaling 436 samples. Table 1 provides a concise overview of the dataset. To rigorously assess the generalization capability of the developed models, a distinct validation strategy was employed. All 316 samples from the literature were used as the training set for model development and hyperparameter optimization. The remaining 120 samples, originating from an independent experimental program conducted by the authors, were reserved as a completely external testing set. This approach ensures that the test data were never seen during the training and optimization phases, providing a stringent and realistic evaluation of the model’s ability to generalize to new data from a different source.

While the compiled dataset provides a comprehensive foundation for this study, it is important to acknowledge its inherent limitations. The total sample size, although substantial for the domain of empirical studies on CRMA, is at the lower bound for training complex machine learning models without encountering the risk of overfitting or reduced generalizability. To mitigate this, strong regularization techniques, early stopping based on validation performance within the GA optimization framework, and the use of inherently robust ensemble methods were employed. Furthermore, the independent external test set provides a crucial, albeit limited, checkpoint for generalization. Future work would greatly benefit from the continuous expansion of such datasets to encompass an even wider range of material sources, curing conditions, and laboratory protocols, which would further enhance model robustness and reliability.

The input features of the dataset encompass key factors governing CRMA pavement performance, including RAP content, curing time, curing temperature, aggregate gradation, filler type, aggregate type, compaction method, and water-cement ratio. All these features have been confirmed to critically impact CRMA pavement performance. Among them, RAP content, curing time, curing temperature, and water-cement ratio are numerical variables, while aggregate gradation, filler type, aggregate type, and compaction method are categorical variables. For instance, curing temperature ranges from 25 °C to 80 °C, and the compaction methods include Marshall compaction, gyratory compaction, vertical vibration compaction, among others. The output indicators center on the core pavement performance metrics of CRMA, namely the high-temperature performance indicator dynamic stability and the low-temperature performance indicator indirect tensile strength.

Table 2 presents the general characteristics of the dataset, including the statistical parameters maximum value (Max), minimum value (Min), mean value (Mean), and coefficient of variation (CV) for each numerical feature. The CV is defined as the ratio of the standard deviation to the mean: a higher CV indicates greater data dispersion and inferior stability, while a lower CV signifies smaller dispersion and better stability. As shown in Table 2, the CV values of all numerical features fall within a reasonable range (0.1677~1.2315). This not only ensures the variability of variables such as curing time and temperature but also guarantees the stability of core parameters such as water-cement ratio and RAP content, making the dataset well-suited for training and validating machine learning models.

**Figure 1:**
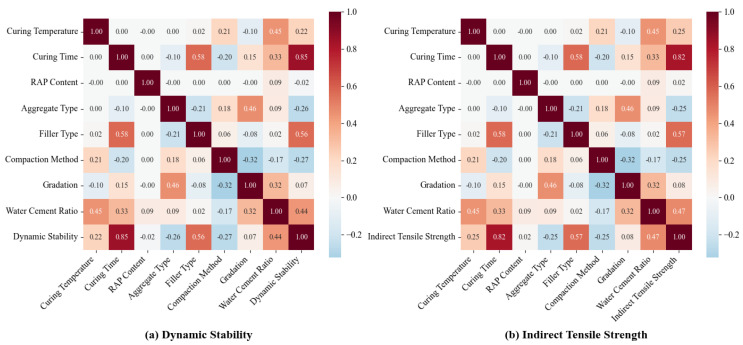
Feature correlation heatmap of input parameters and output variables.

Figure 1 depicts the feature correlation matrix among 8 input variables and 2 output variables, aiming to facilitate a clearer understanding of the sensitivity of each parameter. Specifically, Figure 1a illustrates the correlation relationships with DS, while Figure 1b presents those with ITS. The elements of the matrices are derived from the Pearson correlation coefficient (P), which quantifies the linear correlation between two variables. The value of P ranges from −1 to 1: a value of −1 denotes a perfect negative linear correlation, whereas 1 indicates a perfect positive linear correlation. Excessively strong correlations between input variables (either positive or negative) may result in diminished predictive efficiency for fracture toughness, thereby hindering the ability to isolate the independent influence of individual variables. The correlation coefficients presented in Figure 1 show that all values fall within a reasonable range, far from the extreme values of −1 and 1. These results collectively confirm that all the selected input variables are appropriate for developing the machine learning model.

## 3. Methodology

### 3.1. Data Preprocessing

Prior to model training, the target dataset underwent systematic preprocessing. First, a comprehensive quality assessment was conducted, confirming no missing values, outliers, or logical inconsistencies, which ensured data integrity without the need for imputation or sample exclusion. For numerical features (including curing temperature, curing time, RAP content, water-cement ratio, dynamic stability, and indirect tensile strength), Z-score standardization was applied using the Formula (1):

(1) $$x_{scaled}=\frac{x-\mu }{\sigma }\;$$

where x and $x_{scaled}$ represent the original feature values and the corresponding values after standardization, respectively, μ represents the feature mean and σ denotes the unbiased standard deviation. This resulted in numerical features with a mean centered around a baseline value and a standard deviation within a consistent range, effectively eliminating dimensional biases. Categorical features (including aggregate type, filler type, compaction method, and gradation) were converted to a numerical format via one-hot encoding, with all categories retained to avoid introducing false ordinal relationships. This method provides a dimensionally consistent and reliable input for subsequent machine learning modeling.

### 3.2. Machine Learning Models

**Figure 2:**
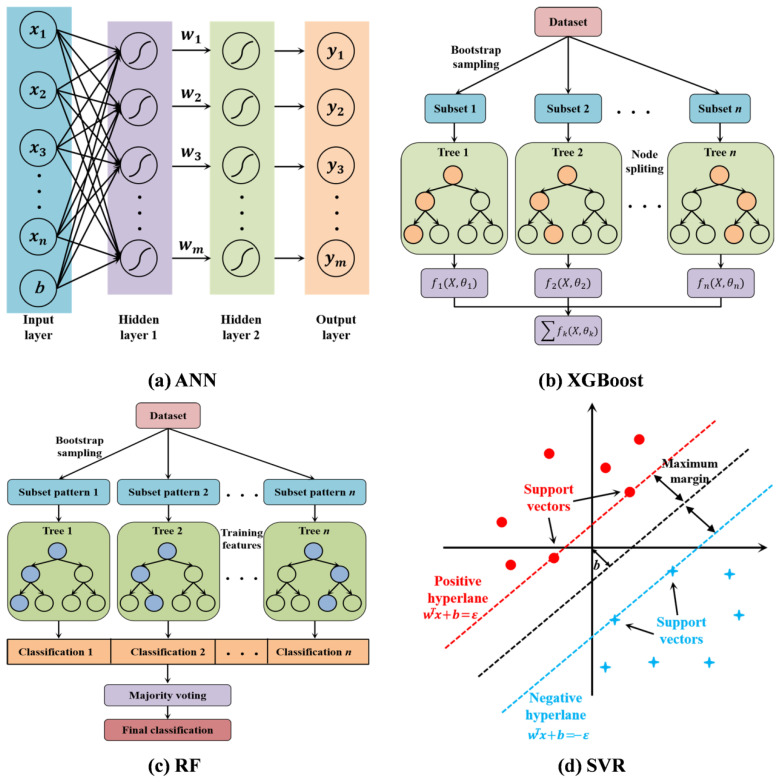
Graphical illustration of four machine learning models.

Machine learning encompasses several primary paradigms with applications spanning classification and regression tasks. For regression problems, supervised learning is particularly effective, as it trains estimators to capture the predictive relationship between independent variables and target variables by leveraging labeled data. In the context of CRMA performance prediction, the road performance indicators exhibit complex nonlinear correlations with multiple influencing factors, including RAP content, curing conditions, aggregate gradation, filler type, and mix asphalt properties. To accurately model these intricate relationships and ensure the reliability of performance predictions, four machine learning models widely validated in road engineering regression tasks were selected: Artificial Neural Network, Extreme Gradient Boosting, Random Forest, and Support Vector Regression. The schematic diagrams of these four models are illustrated in Figure 2, and their respective principles and implementation details are described as follows.

#### 3.2.1. Artificial Neural Network (ANN)

The ANN adopted in this study is a backpropagation neural network model, inspired by the information processing mechanism of the human brain. As illustrated in Figure 2a, it consists of an input layer, one or more hidden layers, and an output layer, where each layer comprises interconnected nodes with weighted connections. The input layer receives feature variables (e.g., RAP content ratio, curing time, gradation parameters), which are transmitted to hidden layers through linear weighting and nonlinear activation functions. A neuron output in the hidden layer is calculated as follows:

(2) $${neu}_{m}=\sum_{i=1}^{n}w_{i}x_{i}+b_{i}$$

where $x_{i}$ is defined as the input signal of input neuron i, $w_{i}$ and $b_{i}$ are donated as the weight and bias between the connection of input and hidden neuron, ${neu}_{m}$ represents the weighted sum produced in neuron m through the activation function. The Hyperbolic Tangent Function (Tanh) is employed in this study as the activation function to introduce nonlinearity, enabling the model to capture complex relationships between input features and CRMA road performance indicators. Tanh activation function is expressed as:

(3) $$Y_{i}=Tanh({neu}_{m})=\frac{e^{{neu}_{m}}-e^{-{neu}_{m}}}{e^{{neu}_{m}}+e^{-{neu}_{m}}}\;$$

Tanh activation function transfer the weighted sum of neuron ${neu}_{m}$ to the output value $Y_{i}$ as the nonlinear shape. The output layer then generates predicted values via the nonlinear activation function for regression tasks. During training, ANN algorithm minimizes the loss function between predicted and actual values by iteratively adjusting connection weights and bias terms. The gradient of the loss function is computed from the output layer to the input layer, and weights are updated using gradient descent optimization. The optimized hyperparameters of weight and bias based on GA algorithm enhance the convergence speed of the model and avoid overfitting, ensuring robust generalization to unseen data. The strength of ANN lies in its ability to model non-linear and interactive relationships without prior assumption of data distribution, making it suitable for predicting CRMA performance influenced by multiple coupled factors.

#### 3.2.2. Extreme Gradient Boosting (XGBoost)

XGBoost is an optimized ensemble learning algorithm characterized by high prediction accuracy and computational efficiency. It constructs a strong learner by sequentially adding weak regression trees, where each new tree is trained to minimize the residual error of the combined model from the previous iteration. XGBoost introduces regularization terms into the loss function to prevent overfitting, and employs a shrinkage learning rate to control the contribution of each weak learner, enhancing model generalization.

The algorithm adopts a greedy strategy to split tree nodes based on the gain of split criteria, and supports parallel computing for feature selection to accelerate training. For CRMA performance prediction, XGBoost processes input features such as filler type, curing temperature to learn hierarchical decision rules, effectively capturing both linear and non-linear relationships. The number of trees, maximum tree depth, subsample ratio, and minimum child weight are optimized based on GA to balance the complexity and performance of XGBoost. These advantages lie in its ability to handle high-dimensional data, resist noise interference, and provide feature importance ranking, facilitating subsequent interpretability analysis.

#### 3.2.3. Random Forest (RF)

RF is an ensemble learning method that integrates multiple independent decision trees to improve prediction accuracy and reduce overfitting. It is constructed through bootstrap sampling to generate diverse training subsets for each tree, and random feature selection to avoid correlation between individual trees. Each decision tree in the forest is trained independently using a Classification and Regression Tree algorithm, and the final prediction result is determined by averaging the outputs of all trees for regression tasks.

For CRMA road performance prediction, RF processes input variables to establish multiple decision trees, each capturing different aspects of the feature-performance relationship. The number of trees, maximum tree depth, minimum samples split, maximum features are optimized based on GA algorithm to control tree complexity and ensemble diversity, preventing overfitting while maintaining predictive power. RF exhibits strong robustness to outliers and missing data, and can quantify feature importance through Gini importance or permutation importance, providing insights into the key factors influencing CRMA performance. Its parallelizable training process and low sensitivity to hyperparameter tuning make it a practical choice for large-scale dataset analysis.

#### 3.2.4. Support Vector Regression (SVR)

SVR is a machine learning algorithm derived from statistical learning theory, designed to find an optimal hyperplane that minimizes prediction error while maximizing the margin of separation. Unlike traditional regression methods that minimize the sum of squared errors, SVR introduces an insensitive loss function, allowing small prediction errors within a tube without penalty, which enhances the model’s generalization ability. For non-linear regression problems, SVR maps input features into a high-dimensional feature space using a kernel function, where a linear hyperplane can be constructed to fit the data.

The RBF kernel is selected in this study for its ability to model complex non-linear relationships without prior knowledge of data distribution. The regularization parameter, kernel coefficient and epsilon are optimized to balance the trade-off between model complexity and prediction accuracy. The regularization parameter controls the penalty for errors outside the tube. Kernel coefficient determines the influence of individual training samples, and epsilon defines the width of the insensitive tube. For CRMA performance prediction, SVR processes input features to construct a non-linear regression model, enabling accurate prediction of rut resistance, low-temperature crack resistance, and other indicators. The strength of SVR allows it more suitable to cope with small sample sizes, and making it complementary to other ensemble and neural network models through structural risk minimization.

### 3.3. Hyperparameter Optimization Using Genetic Algorithm

To enhance the prediction accuracy and generalization ability of the selected machine learning models ANN, XGBoost, RF and SVR, a GA-based hyperparameter optimization framework was developed. GA is a population-based global optimization method inspired by biological evolution principles, iteratively evolves candidate solutions to approximate the global optimum, effectively avoiding local optima that often limit traditional tuning methods [21]. The framework is tailored to the characteristics of each model’s hyperparameters, with key steps detailed as follows:

Each chromosome in the GA population represents a unique combination of hyperparameters for the target model. Encoding schemes are designed based on the discrete or continuous nature of hyperparameters:

ANN: Hyperparameters include the sizes of the hidden layers, L2 Regularization and learning rate.

XGBoost: Optimized hyperparameters include the number of trees, maximum tree depth, learning rate, sub-sample ratio, and minimum child weight. These are encoded as integers or real numbers based on their value ranges.

SVR: Key hyperparameters consist of kernel type, regularization parameter, kernel coefficient, and epsilon number.

RF: Hyperparameters to optimize include the number of trees, maximum depth, minimum samples split, and maximum features.

The fitness function in GA evaluates the performance of each hyperparameter combination, guiding the evolution direction. For all models, fitness is defined using the mean squared error from 5-fold cross-validation on the training dataset, ensuring robust performance estimation. A lower fitness value indicates a superior hyperparameter combination. The optimization process involves iterative evolution of the population through selection, crossover, mutation, and elitism, with the following steps:

(1) Population Initialization: A random initial population of 20 individuals is generated, with each individual corresponding to a hyperparameter combination within predefined ranges. The population size balances diversity and computational efficiency.
(2) Tournament selection is adopted, where 3 individuals are randomly sampled, and the one with the lowest fitness is selected as a parent. This strategy prioritizes high-performance individuals while maintaining population diversity
(3) Crossover: Selected parents undergo crossover with a probability of 0.8 to produce offspring. For continuous parameters, arithmetic crossover is used
(4) Mutation: Offspring are mutated with a probability of 0.02 to enhance exploration. For continuous parameters, Gaussian noise N(0,*σ*) (where *σ* is the standard deviation of the parameter range) is added.
(5) Elitism: The top 5% of individuals (by fitness) are retained in the next generation to preserve high-quality solutions, preventing performance regression.
(6) Termination: The algorithm terminates after 60 generations or when the mean fitness change across 20 consecutive generations is less than the threshold. The optimal hyperparameter combination is selected as the one with the minimum fitness value across all generations.

**Figure 3:**
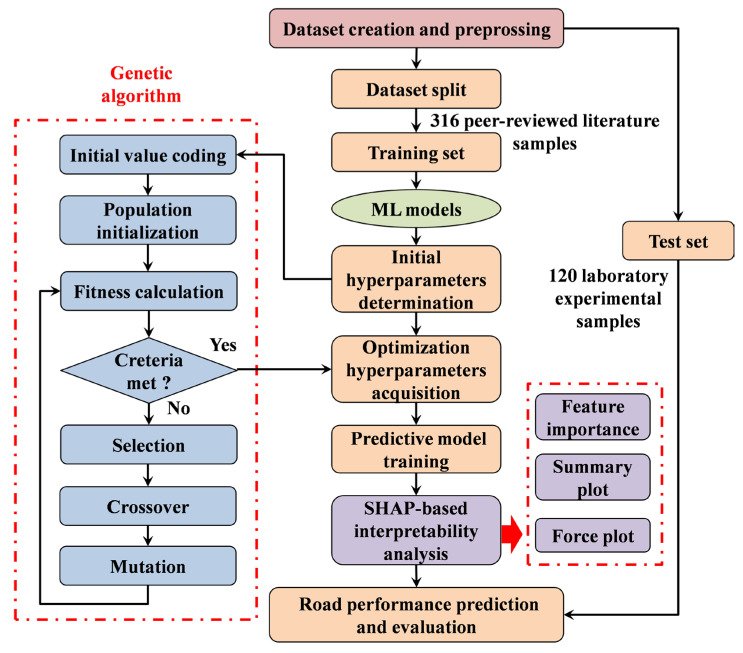
General framework of ML-based road performance prediction for CRMA with GA hyperparameter optimization.

This GA-based framework systematically optimizes hyperparameters for ANN, XGBoost, RF and SVR, ensuring each model operates at its performance frontier for CRMA road performance prediction. The structure of general framework is illustrated as Figure 3.

## 4. Experiment Settings and Performance Assessment

### 4.1. The Hyperparameter Settings and Training Results of GA-Based Optimization

To validate the effectiveness of 4 different ML models on the CRMA road performance prediction, a comprehensive and appropriate training, hyperparameter optimization and evaluation mechanism was built, as illustrated in Figure 3. After preprocessing and normalizing the dataset, a strict source-based data splitting strategy was adopted instead of random assignment: 316 samples from peer-reviewed literature served exclusively as the training set for model development, while 120 samples from original laboratory experiments were reserved as a completely independent external test set. 5-fold cross-validation was implemented on the training set during hyperparameter tuning to enhance evaluation rigor. ANN, XGBoost, RF and SVR models were then trained on the training set with optimal hyperparameters, which were determined via the GA-based framework to boost the predictive accuracy and generalization capability of model, with the test set never involved in any part of the model building process to ensure unbiased generalization assessment.

**Figure 4:**
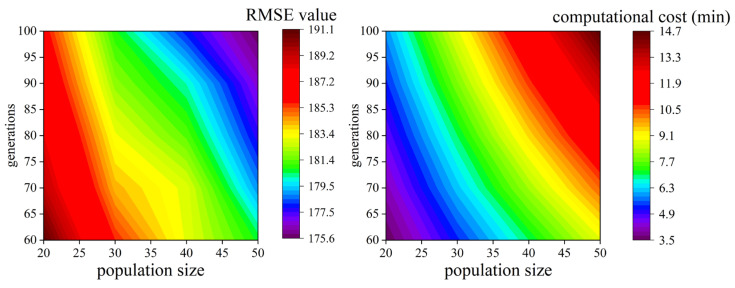
The prediction accuracy and computational cost results of XGBoost model.

To investigate the impact of GA configuration on the prediction accuracy and computational cost of the XGBoost model for CRMA road performance prediction, a systematic sensitivity analysis was conducted and the results are presented in Figure 4. The experiment was designed with varying population sizes (20, 30, 40, and 50 individuals) and evolution generations (60, 70, 80, 90, and 100 generations), and each configuration was repeated 30 times with different random seeds to eliminate the interference of random factors. The prediction accuracy and computational cost results of XGBoost model are presented in Figure 4.

From Figure 4, a limited sensitivity analysis further verified that although the optimal hyperparameter combinations of the top-performing XGBoost model showed minor fluctuations under different random seeds, the model’s performance remained highly stable during 5-fold cross-validation, with coefficient of determination (R^2^) varying by less than ±0.005. This indicates that while the exact optimal hyperparameter point identified by GA may differ slightly, the algorithm can consistently locate the high-performance hyperparameter region. Notably, the GA configuration with 20 individuals and 60 generations was found to stably converge to high-quality prediction solutions while achieving substantially lower computational costs compared to larger population sizes or more generations. For this reason, this specific GA setup was selected as the final optimization configuration for subsequent model training and validation in the study, balancing predictive accuracy and computational efficiency effectively.

To test the performance of 4 ML models, two hyperparameter optimization approaches were employed for comparative analysis: GA optimization and grid search. The grid search method was utilized to exhaustively traverse all hyperparameter configuration combinations defined in the parameter space, serving as a baseline to verify the efficiency of GA-based optimization. For the GA optimization approach, it was configured with a population size of 20 individuals evolving over 60 generations, utilizing tournament selection with a size of 3. The probabilities of blend crossover and Gaussian mutation were set to 0.8 and 0.2, respectively, which enables efficient exploration of the hyperparameter space while maintaining population diversity. The final optimized ANN architecture consisted of an input layer with 8 neurons and an output layer with a single neuron for regression. The Tanh activation function was used in all hidden layers, and the output layer used a linear activation. The model was trained using the Adam optimizer with and employed L2 regularization to prevent overfitting.

The optimization ranges of hyperparameter values and their default values for model training without GA optimization or grid search are presented in Table 3. All experiments were conducted on a laptop equipped with the Windows 10 operating system and an Intel Core i7 CPU (clocked at 2.9 GHz). Each GA optimization experiment can be completed within 5 min, demonstrating the operational feasibility of the proposed method. For both optimization approaches, the fitness function was uniformly defined as R^2^ obtained from 5-fold cross-validation on the training dataset, with the core optimization objective being to maximize this R^2^ value, ensuring consistent evaluation metrics for fair performance comparison between the two methods.

**Figure 5:**
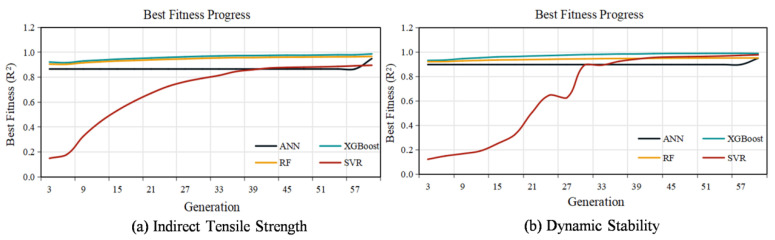
The fitness progress results of 4 ML models with GA-based hyperparameter optimization.

The optimization progress, illustrated in Figure 5, revealed distinct convergence patterns across different models and performance indicators. For Indirect Tensile Strength prediction in Figure 5a, the XGBoost model demonstrated exceptional performance with rapid convergence to near-perfect fitness within the initial 20 generations, indicating efficient parameter space exploration. The ANN and Random Forest models exhibited steady fitness improvements throughout the optimization process, achieving high performance levels by the final generation. The SVR model, while starting from a lower baseline fitness, showed consistent enhancement over generations, reflecting the capability of GA to escape local optima and progressively refine hyperparameter combinations.

For Dynamic Stability prediction in Figure 5b, XGBoost again dominated the optimization landscape, reaching superior fitness values early in the evolutionary process. Both RF and ANN displayed gradual but significant fitness improvements, converging to high-performance regions after approximately 40 generations of optimization. The SVR model demonstrated sustained fitness gains across the entire generational span, ultimately achieving competitive performance through systematic parameter tuning. The comprehensive optimization results underscore the critical importance of GA-driven hyperparameter configuration for maximizing model capabilities in pavement performance prediction.

The systematic hyperparameter optimization enabled each machine learning model to reach its maximum predictive potential, with XGBoost achieving particularly remarkable performance for both road performance indicators. The significant performance gains observed over default parameter configurations validate the necessity of automated optimization approaches for complex machine learning applications in pavement engineering. The genetic algorithm proved effective in navigating high-dimensional parameter spaces and identifying optimal hyperparameter combinations that would be challenging to discover through manual tuning methods.

### 4.2. Performance Evaluation Indicators

To comprehensively and systematically assess the predictive performance of the selected ANN, XGBoost, RF, SVR models for CRMA road performance, four widely used statistical metrics in the field of regression tasks were adopted in this study. The metrics includes R^2^, Root Mean Square Error (RMSE), Mean Absolute Error (MAE), and Mean Absolute Percentage Error (MAPE). These metrics complement each other by capturing different dimensions of prediction errors, enabling a rigorous and multi-faceted evaluation of model efficacy and avoiding one-sided judgments based on a single metric.

R^2^ quantifies the goodness-of-fit between the predicted values and actual values of CRMA road performance indicators. Its value ranges from 0 to 1, with values closer to 1 indicating that the model can explain a larger proportion of the variance in the target variable, reflecting superior explanatory power and fitting effect.

Calculated as the square root of the average of squared differences between predicted and actual values, RMSE is highly sensitive to outlier values due to the squaring of errors. A smaller RMSE indicates a lower overall prediction error, with the metric retaining the same unit as the target variable, facilitating intuitive understanding of error magnitude.

MAE represents the arithmetic mean of absolute differences between predicted and actual values. Unlike RMSE, it is not overly influenced by extreme values, offering robust resistance to outliers and reflecting the average absolute deviation of predictions more directly. A smaller MAE indicates higher prediction accuracy and stability of the model.

MAPE normalizes errors by calculating the average of absolute percentage differences between predicted and actual values. It effectively eliminates the impact of the target variable’s scale, enabling comparisons across different performance indicators. MAPE ranges from 0 to 1 (or 0% to 100%), with smaller values indicating higher relative prediction accuracy, and it reduces the interference of individual extreme values on the overall error assessment.

(4) $$R^{2}=1-\frac{\sum_{i=1}^{n}{\left(y_{i}-{\hat{y}}_{i}\right)}^{2}}{\sum_{i=1}^{n}{\left(y_{i}-\bar{y}\right)}^{2}}$$

(5) $$RMSE=\sqrt{\frac{1}{n}\sum_{i=1}^{n}{\left(y_{i}-{\hat{y}}_{i}\right)}^{2}}$$

(6) $$MAE=\frac{1}{n}\sum_{i=1}^{n}\left|y_{i}-{\hat{y}}_{i}\right|$$

(7) $$MAPE=\frac{1}{n}\sum_{i=1}^{n}\left|\frac{y_{i}-{\hat{y}}_{i}}{y_{i}}\right|\times 100\%$$

For all metrics, the evaluation criteria are consistent: lower values of RMSE, MAE, and MAPE, coupled with a R^2^ value closer to 1, indicate better predictive accuracy and generalization ability of the model. In Equations (4)–(7) for these metrics, $y_{i}$ denotes the actual value of the i-th sample, ${\hat{y}}_{i}$ represents the corresponding predicted value, $\bar{y}$ is the mean of the actual values, and n is the total number of samples in the test set.

### 4.3. Model Interpretability Analysis Based on Shapley Additive Explanations (SHAP)

To address the “black box” limitation of the selected ANN, XGBoost, RF, SVR models and quantitatively identify key factors influencing CRMA road performance, SHAP method is adopted for systematic interpretability analysis. As a mathematically rigorous framework, SHAP overcomes the subjectivity of traditional feature importance evaluation by quantifying the marginal contribution of each input feature to individual predictions, enabling both global and local insights into model decision-making.

The core principle of SHAP lies in calculating the Shapley value for each feature, which measures the deviation between the prediction of model for a specific sample and the baseline value through weighted analysis of all possible feature subsets. This ensures each feature is assigned a fair and unique importance score. The Shapley value $\phi_{i}$ for the i-th feature is formally defined as:

(8) $$\phi_{i}=\sum_{S\subseteq M\backslash \{i\}}\frac{\left|S\right|!(P-\left|S\right|-1)!}{P!}[h_{x}(S\cup \{i\})-h_{x}(S)]$$

where M denotes the complete set of input features, P is the total number of features, S represents arbitrary subsets of features excluding the i-th feature, and $h_{x}(.)$ denotes the prediction function of the model conditional on the given feature subset. A positive $\phi_{i}$ indicates the feature promotes an increase in the target performance indicator, while a negative value signifies an inhibitory effect; the absolute value reflects the magnitude of the influence of the feature.

## 5. Results and Discussion

### 5.1. Prediction Performance on Dynamic Stability

Table 4 presents the comprehensive predictive performance of four machine learning models for DS prediction under three hyperparameter configurations: genetic algorithm (GA) optimization, default hyperparameters, and grid search. Overall, GA-optimized models outperformed their default-parameter counterparts across all evaluation metrics, and exhibited competitive accuracy relative to grid search while achieving superior computational efficiency.

The XGBoost model exhibited exceptional predictive capability after GA optimization, achieving an R^2^ value of 0.9694, representing a 2.60% improvement over its default parameter configuration. More notably, the error metrics showed remarkable reductions, with RMSE decreasing from 277.29 to 191.06 (with 31.10% improvement), MAE improving from 214.66 to 166.70, and MAPE reducing from 5.80% to 4.05%. While grid search-tuned XGBoost achieved a marginally higher R^2^, the performance gap between GA optimization and grid search was negligible, indicating that GA could capture near-optimal hyperparameter combinations for XGBoost. These enhancements demonstrate that GA optimization effectively captured the complex nonlinear relationships between input features and dynamic stability. From another perspective, the superior performance of optimized models can be attributed to their ability to accurately represent the intricate mechanisms governing the high-temperature stability of CRMA. The dynamic stability, which reflects the mixture’s resistance to permanent deformation under repeated loading, is fundamentally determined by the internal friction among aggregate particles and the cohesion provided by the emulsified asphalt matrix. The optimized models successfully captured how these fundamental properties are influenced by compositional factors and curing conditions.

The SVR model showed the most dramatic performance improvement through optimization, with R^2^ increasing from 0.8723 to 0.9558 (with 8.74% relative improvement) and RMSE decreasing by 50.26% from 483.66 to 240.57. This substantial enhancement indicates that the GA successfully navigated the complex parameter space of SVR, optimizing the regularization parameter $C_{R}$, kernel coefficient γ, and epsilon-tube parameter ε to balance model complexity and prediction accuracy. The improved SVR performance particularly benefited from better handling of the nonlinear interactions between RAP characteristics and emulsified asphalt properties, which critically affect the formation of a continuous asphalt film around aggregates, which is a key factor in determining rutting resistance. ANN and RF models also demonstrated meaningful improvements, with R^2^ values increasing by 3.23% and 2.83%, respectively. The consistent performance gains across all model types validate the effectiveness of the GA framework in identifying optimal hyperparameter combinations that would be challenging to discover through manual tuning methods.

The computational expense of the GA optimization is non-negligible and depends on the model complexity, population size, number of generations, and the cost of evaluating a single hyperparameter set. Table 5 quantifies the computational cost of the three hyperparameter configurations in minutes for DS prediction, revealing a clear trade-off between performance tuning methods and time efficiency. The ML models with default values achieved the lowest computational overhead among the three approaches for all models, whereas grid search incurred drastically higher time costs despite marginal accuracy gains. For XGBoost, the GA-based hyperparameter tuning only required 1.12 min, which was 67.14% lower than the default parameter training time and 97.86% less than the grid search time. Similarly, the GA-optimized RF model completed tuning in 0.87 min, a 78.03% reduction compared to grid search and a 78.03% decrease relative to default parameter training. Even for computationally intensive models like ANN and SVR, GA optimization maintained its efficiency advantage: the GA tuning time of ANN was 95.20% shorter than grid search, and the GA time of SVR was 92.73% less than grid search. The efficiency of GA stems from its population-based global search mechanism, which avoids the exhaustive parameter enumeration of grid search, balancing prediction accuracy and computational resource consumption for pavement engineering applications with limited computing budgets.

**Figure 6:**
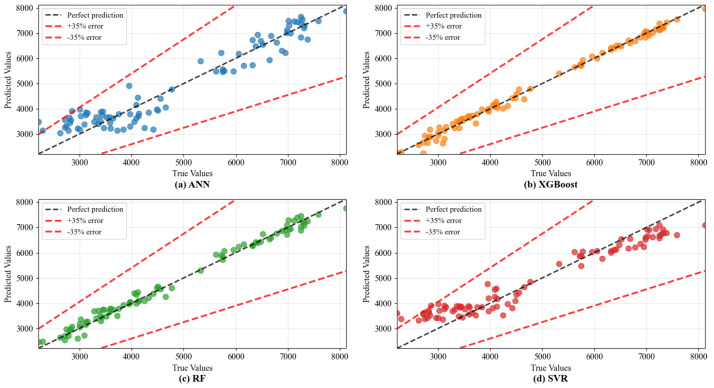
The comparison between experimental and predicted values of ML models on dynamic stability.

**Figure 7:**
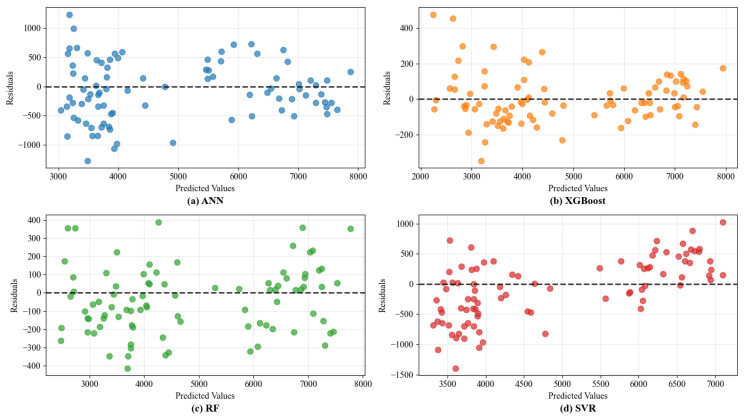
The residual plots of predicted values of ML models on dynamic stability.

The visual analysis in Figure 6 further corroborates these quantitative findings. The prediction points generated by the GA-optimized models, especially XGBoost and SVR, align more closely with the line of perfect prediction compared to their default counterparts, demonstrating reduced systematic bias. Notably, the SVR model shows the most dramatic visual improvement, with its predictions becoming tightly clustered after optimization. The residual plots in Figure 7 provide deeper insight into error distribution. The residuals for the optimized models are more homogeneously distributed around zero across the entire range of predicted values, indicating no strong heteroscedasticity. This homogeneous scatter suggests the models have successfully captured the underlying relationships without overfitting to specific performance ranges. The remaining unexplained variance is likely attributable to inherent experimental variability and factors not explicitly encoded in the dataset, such as subtle variations in compaction energy within the same nominal method or RAP source variability.

### 5.2. Prediction Performance on Indirect Tensile Strength

Table 6 outlines the predictive performance of the four ML models for ITS prediction across the three hyperparameter settings, mirroring the DS prediction trend with GA optimization yielding significant accuracy gains over default parameters and competitive results against grid search. While grid search can marginally outperform GA in individual metrics for certain models, it comes at the cost of drastically higher computational overhead as documented in Table 7. XGBoost again demonstrated superior performance under GA optimization with an R^2^ of 0.9643, though the performance gap between optimized and default parameters was less pronounced compared to dynamic stability prediction; notably, grid search-tuned XGBoost achieved a slightly higher R^2^ of 0.9682 and a lower MAPE of 3.96%, yet its computational time was nearly 48 times that of GA optimization, making GA a more practical choice for engineering applications with limited computing resources. The ANN model showed the most significant improvement in indirect tensile strength prediction under GA optimization, with R^2^ increasing from 0.8968 to 0.9331 and RMSE decreasing by 20.19% from 0.0822 to 0.0656. This performance was nearly on par with grid search-tuned ANN, but GA completed the tuning process in just 1.70 min, whereas grid search required 35.28 min. It highlights the efficiency in capturing optimal network architecture and learning parameters without sacrificing predictive precision. This improvement suggests that the GA optimization effectively identified optimal network architecture and learning parameters that enhanced the ability of the model to capture the underlying patterns in the data. The tensile strength of CRMA is governed by complex interfacial bonding between RAP particles, virgin aggregates, and emulsified asphalt, which involves both mechanical interlocking and physicochemical adhesion mechanisms. The optimized ANN better represented these complex interactions, particularly the role of cement hydration products in forming a reinforced network structure that enhances tensile resistance, and it achieved this capability with far less computational investment than grid search.

XGBoost maintained its leading position with the highest R^2^ and lowest error metrics among all models under GA optimization, achieving an RMSE of 0.0507, MAE of 0.0393, and MAPE of 5.48%. Its performance was only marginally surpassed by its grid search counterpart, while avoiding the prohibitive time cost of gird search method. The RF model performed competitively under GA tuning, with R^2^ = 0.9576, closely following XGBoost in prediction accuracy; in contrast, grid search-tuned RF only reached an R^2^ of 0.9479, even with its much longer tuning duration, further validating the superiority of GA in balancing model performance and computational efficiency for tree-based models. It should be noted that the dynamic range of the ITS dataset in this study is limited to 0.91 MPa, from 0.45 to 1.36 MPa, where high R^2^ might be achieved by mean-value fitting rather than capturing true variable-performance correlations. Thus, we prioritize absolute error metrics such as RMSE, MAE for evaluating engineering validity. For the GA-optimized XGBoost model, the RMSE accounts for merely 5.57% of the total ITS range, while the MAE corresponds to 4.32% of this range. Both values are substantially below the 10% threshold for engineering-acceptable prediction errors. This confirms that the predictive capability of model originates from capturing the specific effects of individual influencing factors, rather than from simplistic mean-value regression that lacks practical engineering significance. The strong performance of tree-based models for tensile strength prediction can be attributed to their ability to handle the categorical variables representing aggregate type, filler type, and compaction method, which significantly influence the microstructure development and consequent mechanical properties of CRMA, and GA optimization amplified this advantage without the inefficiency of exhaustive grid search parameter enumeration.

The combined analysis of Table 4, Table 5, Table 6 and Table 7 reveals that GA-optimized XGBoost is the optimal model for CRMA road performance prediction, balancing high predictive accuracy and minimal computational cost. GA optimization addresses the dual limitations of default parameters in suboptimal accuracy and grid search in prohibitive computational cost, enabling ML models to efficiently capture the complex relationships between the material-curing factors of CRMA and its road performance. For practical engineering applications, this framework provides a cost-effective and accurate tool for CRMA mix design and performance pre-evaluation, facilitating sustainable pavement maintenance.

**Figure 8:**
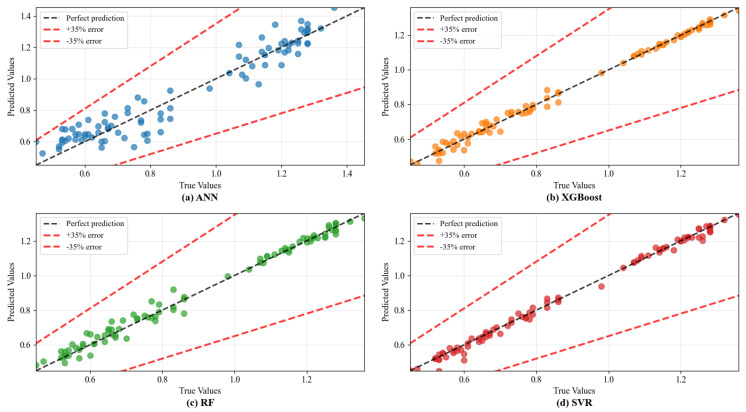
The comparison between experimental and predicted values of ML models on indirect tensile strength.

Figure 8 provides a visual confirmation of the quantitative findings presented in Table 5, depicting the relationship between the experimentally measured and model-predicted ITS values. The GA-optimized XGBoost model demonstrates the most favorable performance, characterized by the tightest clustering of data points along the line of perfect prediction across the entire strength range of 0.45 to 1.36 MPa. This minimal scatter and excellent alignment visually corroborate its superior statistical metrics, with the highest R^2^ and the lowest RMSE. The data indicate a robust capture of the underlying functional mapping from the input features to ITS, effectively generalizing across the diverse mixture compositions in the dataset. In contrast, the predictions from the optimized ANN and Random Forest models, while still showing strong overall agreement, exhibit slightly greater dispersion, particularly noticeable in the higher strength regime. This increased variance could be attributed to two interrelated factors: first, a relative sparsity of high-strength training samples, limiting the models’ ability to precisely learn the relationships specific to this performance region; and second, the potentially increased complexity or different governing mechanisms at higher strength levels, which may be more challenging for certain model architectures to encapsulate without overfitting.

**Figure 9:**
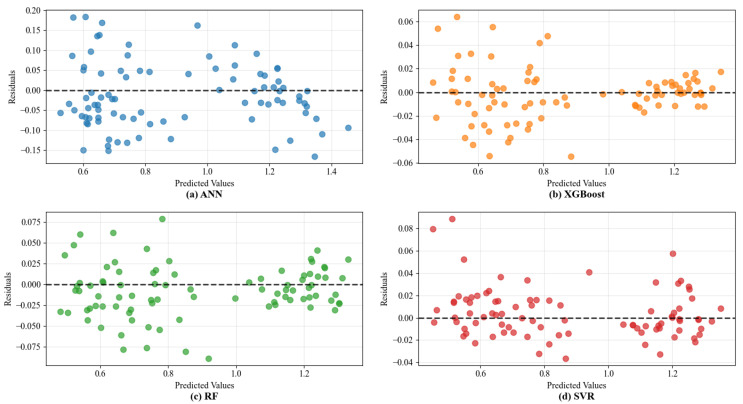
The residual plots of predicted values of ML models on indirect tensile strength.

The residual plots in Figure 9 offer a more nuanced diagnostic perspective, crucial for assessing model reliability and identifying areas for improvement. For ITS prediction, the GA-optimized XGBoost model exhibits the most desirable pattern: a homogeneous, random-like distribution of residuals around the zero-error line across the entire range of predicted values. The absence of any clear trend and the consistent variance suggest that the model has successfully learned the fundamental relationships without introducing significant systematic bias or overfitting to particular subsets of the data. This pattern instills confidence in its general applicability. Conversely, the SVR model, despite showing marked improvement after optimization, reveals a discernible pattern of systematic underestimation for samples with predicted ITS values in the intermediate range. This indicates a localized model bias, suggesting that the selected RBF kernel and optimized hyperparameters, while effective globally, may not perfectly capture the specific nonlinearities governing this performance segment. This presents a clear avenue for future refinement, such as exploring alternative kernel functions or explicit feature engineering for interactions prevalent in mid-strength mixtures. The slight curvature in the residual plot for SVR suggests that incorporating interaction terms or using a different kernel might further improve its performance.

Furthermore, the encoding of compaction methods as categorical variables assumes homogeneity within each method label (e.g., “Marshall”). In reality, variations in compaction energy, hammer weight, or number of blows between different experimental protocols could introduce unaccounted-for variance. This source of noise may contribute to the unexplained residuals observed in Figure 7 and Figure 9. In Figure 7 and Figure 9, it can be observed that although the residuals are generally distributed around the zero line, there are intervals of predicted values where the residuals exhibit greater dispersion or slight clustering. This variability may well be attributed to the mechanism described above. For instance, in Figure 7, data points with predicted values around 4000~5000 times/mm show a relatively wide scatter of residuals. This likely includes samples that all belong to either the “rotary compaction” or “vertical vibration compaction” category but display varied performance due to differences in specific process parameters. The model fails to distinguish among these subtle variations, resulting in systematic scattered errors in predictions. Future studies would benefit from recording and using continuous parameters like compaction energy (kJ/m^3^) or achieving density (%) as model inputs, which would more precisely capture the effect of compaction on the resulting mixture structure and performance.

The physical interpretation of these findings relates to the fundamental mechanisms controlling tensile strength development in CRMA. The accurate prediction of indirect tensile strength requires capturing the complex interplay between aggregate interlock, mastic cohesion, and interfacial bonding strength. The superior performance of optimized models, particularly XGBoost, suggests they effectively represent how these mechanisms are influenced by factors such as curing conditions, mixture composition, and compaction methods. The random residual distribution further indicates that the models have generalized well across different failure mechanisms, including adhesive failure at asphalt-aggregate interfaces and cohesive failure within the mastic phase.

The comparative analysis between default and optimized parameters reveals that GA optimization provided particular benefits for models with more complex hyperparameter spaces. The performance improvements were more substantial for ANN and SVR, which have multiple interacting parameters, compared to tree-based models that are generally more robust to parameter variations. This finding has practical implications for model selection in CRMA performance prediction, suggesting that with proper optimization, more complex models can achieve superior accuracy despite their increased parameter sensitivity.

### 5.3. Model Interpretation and Feature Importance Analysis

**Figure 10:**
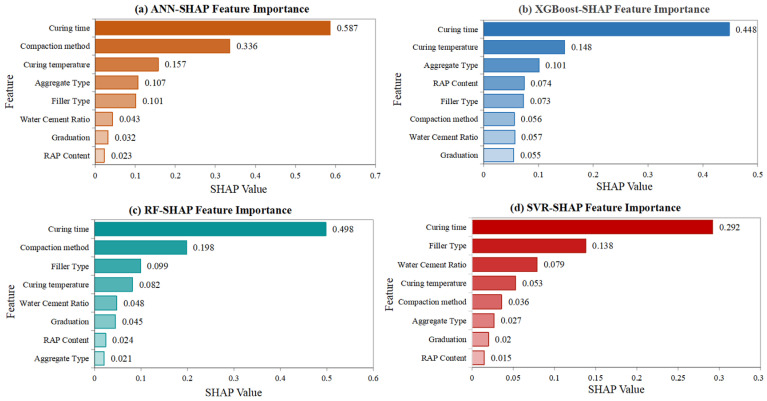
Feature importance ranking of input variables on dynamic stability.

The SHAP analysis provided crucial insights into the relative importance of different input features and their directional effects on CRMA performance indicators. Figure 10 presents the feature importance ranking for dynamic stability prediction, revealing that curing temperature, RAP content, and curing time emerged as the most influential factors across all machine learning models.

For dynamic stability, curing temperature demonstrated the strongest positive correlation, with higher temperatures generally leading to improved stability values. This finding aligns with the fundamental understanding that elevated temperatures accelerate the demulsification process and promote stronger bonding between asphalt and aggregates. The temperature effect is particularly crucial in cold recycling technology, where sufficient heat is essential for complete water evaporation and proper development of asphalt-aggregate adhesion. The optimized models accurately captured the optimal temperature range of 60~80 °C, beyond which excessive temperatures may cause premature asphalt aging.

RAP content showed a complex nonlinear relationship, with optimal performance observed at intermediate levels (70~80%), while excessive RAP content negatively affected dynamic stability due to limitations of aged asphalt. This behavior reflects the dual role of RAP materials: they provide valuable aggregate skeleton but introduce stiffened aged asphalt that may compromise the mixture’s flexibility and bonding characteristics. The SHAP analysis revealed that the optimal RAP content depends on the specific gradation and the properties of the virgin emulsified asphalt, highlighting the importance of balanced mixture design.

Curing time exhibited a strong positive effect on dynamic stability in Figure 10, consistent with the time-dependent nature of strength development in emulsified asphalt mixtures. The extended curing allows for complete breaking of the emulsion, gradual water evaporation, and continued development of cement hydration products when cement is used as filler. The models successfully captured the rapid strength gain during the first 7~14 days followed by more gradual improvement, providing valuable guidance for determining the appropriate curing period before opening to traffic.

**Figure 11:**
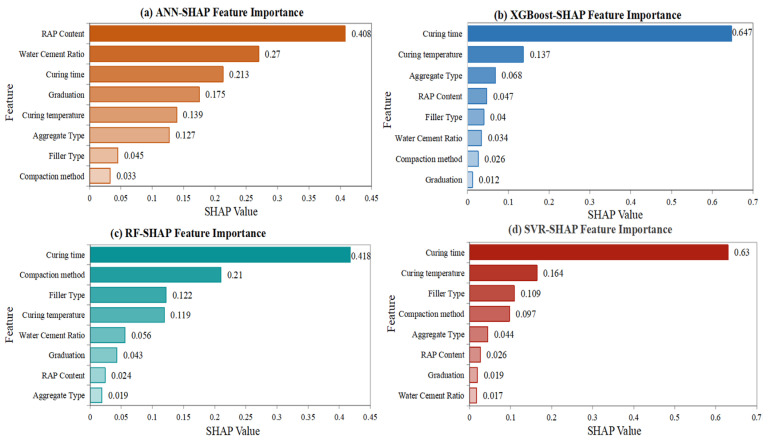
Feature importance ranking of input variables on indirect tensile strength.

In the indirect tensile strength analysis in Figure 11, water-cement ratio and curing time emerged as dominant factors. The water-cement ratio exhibited a negative correlation with tensile strength, consistent with concrete technology principles where excessive water content leads to increased porosity and reduced mechanical strength. In CRMA applications, optimal water content is critical for achieving proper coating of aggregates without creating excessive voids or weakening the mastic structure. The models identified the optimal water-cement ratio range of 0.35~0.45, which ensures adequate workability while maintaining sufficient density and strength.

Curing time demonstrated a strong positive effect on indirect tensile strength, emphasizing the importance of sufficient time for complete demulsification and strength development. The progressive strength gain with extended curing reflects the continued evaporation of residual water and the development of stronger interfacial bonds between constituent materials. This finding supports the current practice of extended curing periods for cold recycled mixtures before subjecting them to significant traffic loads.

**Figure 12:**
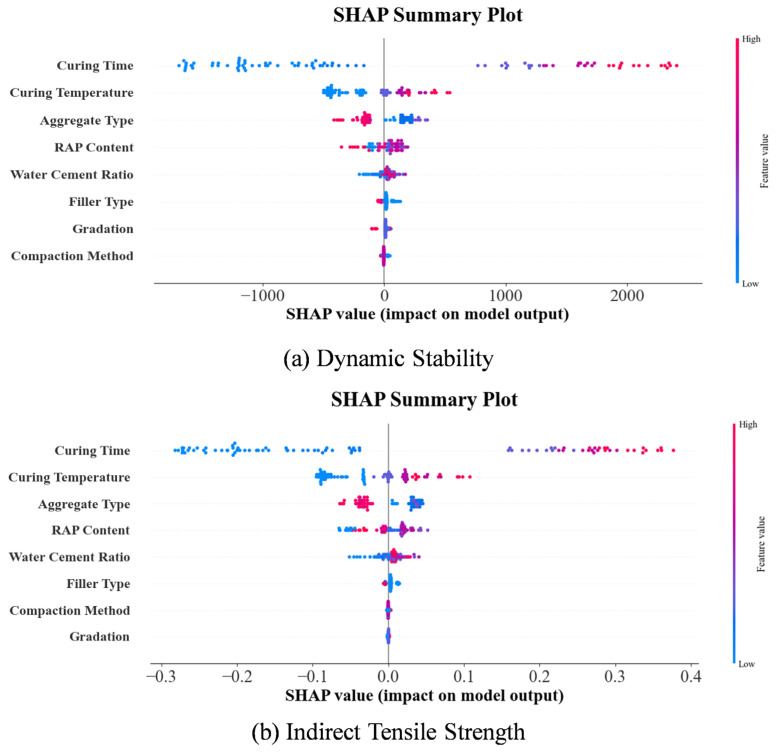
SHAP analysis summary plots of ML models regarding the road performance of CRMA.

**Figure 13:**
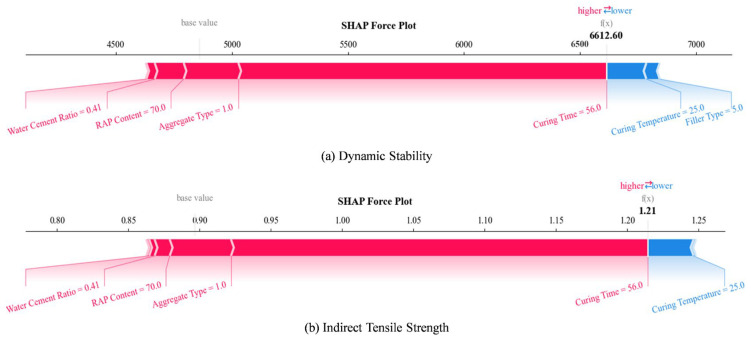
SHAP force plots of ML models regarding the road performance of CRMA.

**Figure 14:**
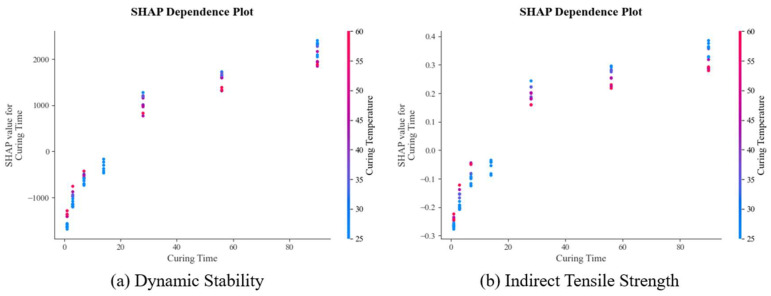
SHAP dependence plots of ML models regarding the road performance of CRMA.

The SHAP summary plots in Figure 12 provide detailed visualization of feature effects, showing how each variable contributes to individual predictions. The force plots in Figure 13 offer local interpretability, illustrating how specific feature combinations influence particular predictions. These interpretability analyses bridge the gap between data-driven predictions and mechanistic understanding, providing practical guidance for mixture design optimization. To explore whether distinct mechanisms govern low-strength vs. high-strength mixtures, a post hoc analysis was conducted by clustering samples based on DS and ITS values and examining SHAP dependence plots for key features within each cluster, which is illustrated in Figure 14. While trends were consistent, the magnitude of feature effects sometimes varied. This suggests that while a single global model captures the dominant relationships efficiently, a future mixture-of-experts approach, where a classifier directs samples to specialized sub-models, could potentially yield further accuracy gains, especially as datasets grow larger and more diverse.

Notably, the feature importance patterns showed some variation between different ML models, suggesting that each algorithm captures slightly different aspects of the underlying relationships. However, the overall consistency in identifying key factors enhances confidence in the robustness of the findings. The identification of curing conditions and mixture composition as dominant factors aligns well with established knowledge in asphalt technology, while the quantitative assessment of their relative importance provides new insights for optimizing CRMA design.

The practical implications relevant to CRMA mix design can be summarized as follows, with the SHAP analysis providing clear quantitative guidance for the optimization of CRMA mix designs. Practitioners should aim for: (1) a curing temperature between 60 °C and 80 °C to maximize the rate of strength development without risking asphalt aging; (2) an RAP content in the range of 70~80% to balance the benefits of recycled material with the need for sufficient virgin binder-aggregate interaction; (3) a minimum curing time of 7~14 days for significant strength gain, with longer periods yielding further improvement for critical applications; (4) a strict control of the water–cement ratio between 0.35 and 0.45 to ensure adequate workability and cement hydration while minimizing porosity. These parameter ranges, derived from the data-driven models, can serve as initial targets in a balanced mix design process, which should still be validated by laboratory performance tests for specific material sources and project requirements.

## 6. Conclusions

Accurately predicting the road performance of CRMA is crucial for its optimal design and sustainable application. This study developed and comprehensively evaluated four machine learning models for predicting the road performance of cold recycled mix asphalt, with particular focus on genetic algorithm optimization of hyperparameters and model interpretability through SHAP analysis. The main conclusions are as follows:

(1) Genetic algorithm optimization significantly enhanced the predictive performance of all machine learning models, with XGBoost emerging as the most effective algorithm for both dynamic stability and indirect tensile strength prediction. The optimized XGBoost model achieved test R^2^ values of 0.9793 for dynamic stability and 0.9694 for indirect tensile strength, representing substantial improvements over its default parameter configuration. This systematic hyperparameter tuning enabled all models to achieve performance levels, particularly in error reduction that would be challenging to obtain through manual parameter selection.

(2) The optimization process demonstrated distinct convergence patterns across different models. XGBoost showed rapid convergence to near-optimal performance within the initial 20 generations, while other models like ANN and Random Forest exhibited more gradual improvement throughout the 60-generation evolutionary process. This efficiency in parameter space exploration makes XGBoost particularly suitable for complex pavement engineering applications where computational resources may be limited.

(3) Comparative analysis between optimized and default parameter configurations revealed substantial performance improvements, particularly for SVR and ANN models. The SVR model showed the most dramatic relative improvement, with its R^2^ for dynamic stability increasing by 7.8% from 0.8973 to 0.9674, while the ANN model showed a 3.65% improvement. These gains highlight the critical importance of proper hyperparameter tuning in machine learning applications for pavement engineering, especially for dynamic stability prediction where complex nonlinear relationships between material composition and performance indicators require sophisticated model configurations.

(4) SHAP-based interpretability analysis quantitatively identified curing temperature, with mean absolute SHAP values ranging from 0.22 to 0.41, RAP content, and curing time as the most influential factors for dynamic stability. For indirect tensile strength, water-cement ratio, with mean absolute SHAP values between 0.18 and 0.35, and curing time dominated the predictions. These findings provide mechanistic insights that align with materials science principles and offer practical guidance for mixture design optimization, revealing optimal ranges for key parameters: curing temperature of 60~80 °C, RAP content of 70–80%, and water-cement ratio of 0.35–0.45.

(5) The research demonstrates that the integration of genetic algorithm optimization with machine learning models creates a powerful framework for CRMA performance prediction, combining high accuracy with computational efficiency and interpretability. The proposed approach represents a significant advancement over traditional empirical methods and provides valuable tools for pavement construction and maintenance.

Future research should focus on expanding the dataset to include more diverse mixture designs, investigating hybrid optimization approaches that combine GA with local search methods. Exploring the integration of physical knowledge into data-driven models could also enhance their generalization capability and physical consistency. Additionally, long-term performance prediction incorporating aging effects and environmental factors would further extend the practical applicability of the developed models. Future study will also envision the integration of multi-modal data sources to enrich predictive models. For instance, computer vision techniques applied to images of pavement surfaces or aggregate gradation could provide high-dimensional input features related to morphology and distress. Advanced architectures like DeepLab [75] and EfficientNet [76] could be leveraged to automate feature extraction from visual data, creating a more comprehensive digital twin of pavement materials for performance prediction.

## Figures and Tables

**Table 1:** Brief overview of sample features in the first 5 rows of the dataset.

ID	Curing Temperature (°C)	Curing Time (Days)	RAP Content (%)	Aggregate Type	Filler Type	Compaction Method	Gradation	W-C Ratio	DS (Times/mm)	ITS (MPa)
1	25	1	60	Limestone	Cement	Marshall	Fine-Graded (AC-13)	0.35	3010	0.58
2	15	1	100	Limestone	Cement	Marshall	Fine-Graded (AC-13)	0.44	2440	0.49
3	60	28	75	Andesite	Lime	Vertical Vibration	Coarse-Graded (AC-25)	0.54	6180	1.13
4	25	56	65	Quartzite	Fly Ash	Rotary	Open-Graded	0.58	5480	1.05
5	35	28	70	Diabase	Lime	Vertical Vibration	Coarse-Graded (AC-25)	0.37	6450	1.19

**Table 2:** Statistic Characteristics of input features and outputs in dataset.

Feature	Feature Type	Max	Min	Mean	CV
Input					
Curing temperature (°C)	Numeric	80	25	45.6	0.556
Curing time (days)	Numeric	90	1	24.8	1.232
RAP content (%)	Numeric	100	50	75.0	0.189
Water Cement Ratio	Numeric	0.62	0.29	0.45	0.168
Aggregate Type	Category	0-Limestone, 1-Basalt, 2-Granite, 3-Diabase, 4-Andesite, 5-Quartzite, 6-Gneiss, 7-Recycled Concrete			
Filler Type	Category	0-Cement, 1-Lime, 2-Fly Ash, 3-Rice Husk Ash, 4-Cement Kiln Dust, 5-Coal Waste Ash, 6-Palm Oil Fuel Ash, 7-Silica Fume, 8-Stone Powder			
Compaction Method	Category	0-Marshall, 1-Gyratory, 2-Vertical Vibration, 3-Rotary, 4-Impact, 5-Kneading, 6-Static			
Gradation	Category	0-Fine-Graded (AC-13), 1-Medium-Graded (AC-20), 2-Coarse-Graded (AC-25), 3-Open-Graded, 4-Dense-Graded, 5-Gap-Graded, 6-SMA			
Output					
Dynamic Stability (times/mm)	Numeric	8120	2180	4849.1	0.310
Indirect Tensile Strength (MPa)	Numeric	1.36	0.45	0.896	0.282

**Table 3:** Hyperparameter Search Ranges for Genetic Algorithm Optimization.

Model	Hyperparameter	Description	Search Range	Default Value
ANN	$\left(H_{1},H_{2}\right)$	Hidden Layer Sizes	(10–200, 5–100)	(100, 50)
	α	L2 Regularization	0.0001–0.1	0.0001
	η	Learning Rate	0.001–0.1	0.001
XGBoost	$N_{est}$	Number of Trees	50–500	100
	D	Maximum Depth	3–15	6
	λ	Learning Rate	0.01–0.3	0.3
	S	Sub-sample Ratio	0.5–1.0	1.0
	$C_{W}$	Minimum Child Weight	1–10	1
SVR	$C_{R}$	Regularization Parameter	0.1–100	1.0
	γ	Kernel Coefficient	0.001–1	0.01
	ε	Epsilon-tube	0.01–0.5	0.1
RF	$N_{est}$	Number of Trees	50–500	100
	$D_{Max}$	Maximum Depth	3–20	5
	$S_{Min}$	Minimum Samples Split	2–20	2
	$F_{Max}$	Maximum Features	0.1–1.0	0.5

**Table 4:** Precision comparison of ML models on dynamic stability.

Parameter Settings	ML Model	R^2^	RMSE	MAE	MAPE
Hyperparameter optimized by GA	ANN	0.9263	299.60	285.64	6.64%
	XGBoost	0.9694	191.06	166.70	4.05%
	RF	0.9385	282.31	252.29	6.20%
	SVR	0.9558	240.57	203.50	4.98%
Hyperparameter with default values	ANN	0.8964	472.16	412.32	10.39%
	XGBoost	0.9448	277.29	214.66	5.80%
	RF	0.9119	302.47	296.59	6.97%
	SVR	0.8723	483.66	432.33	11.15%
Hyperparameter with grid search	ANN	0.9303	281.48	269.40	6.41%
	XGBoost	0.9741	174.38	150.58	3.87%
	RF	0.9434	265.94	236.51	5.91%
	SVR	0.9594	223.91	186.48	4.63%

**Table 5:** Computational cost comparison of ML models on dynamic stability.

Computational Cost (Min)	Hyperparameter Optimization Method		
	GA	Default Values	Grid Search
ANN	1.71	4.80	35.60
XGBoost	1.12	3.53	52.30
RF	0.87	3.96	21.50
SVR	2.89	5.72	39.74

**Table 6:** Precision comparison of ML models on indirect tensile strength.

Parameter Settings	ML Model	R^2^	RMSE	MAE	MAPE
Hyperparameter optimized by GA	ANN	0.9331	0.0656	0.0499	6.76%
	XGBoost	0.9643	0.0507	0.0393	5.48%
	RF	0.9576	0.0523	0.0406	5.81%
	SVR	0.9346	0.0709	0.0536	7.52%
Hyperparameter with default values	ANN	0.8968	0.0822	0.0630	8.43%
	XGBoost	0.9470	0.0554	0.0450	5.97%
	RF	0.9407	0.0611	0.0485	6.60%
	SVR	0.9353	0.0686	0.0519	7.20%
Hyperparameter with grid search	ANN	0.9342	0.0655	0.0499	6.31%
	XGBoost	0.9682	0.0507	0.0392	3.96%
	RF	0.9479	0.0523	0.0405	5.82%
	SVR	0.9647	0.0707	0.0536	4.75%

**Table 7:** Computational cost comparison of ML models on indirect tensile strength.

Computational Cost (Min)	Hyperparameter Optimization Method		
	GA	Default Values	Grid Search
ANN	1.70	4.87	35.28
XGBoost	1.09	3.50	52.34
RF	0.91	3.91	21.12
SVR	2.81	5.69	39.27

## Data Availability

The original contributions presented in the study are included in the article, further inquiries can be directed to the corresponding author.
